# Impact of the Addition of Daratumumab to the Frontline Treatment of Patients with Immunoglobulin Light-Chain Amyloidosis: A Single-Centre Experience

**DOI:** 10.3390/cancers17091440

**Published:** 2025-04-25

**Authors:** Elena Alejo, Cristina Agulló, Borja Puertas, Rocío Eiros, Beatriz Rey-Búa, Carmen Barnés, Marta Rodríguez-González, Lucía López-Corral, Ángel Santos-Briz, Fernando Escalante, María-Luisa Pérez-García, Silvia Jiménez-Cabrera, Norma C. Gutiérrez, Noemi Puig, María-Victoria Mateos, Verónica González-Calle

**Affiliations:** 1 Hematology Department, University Hospital of Salamanca, Cancer Research Center-IBMCC (USAL-CSIC), Instituto de Investigación Biomédica de Salamanca (IBSAL), CIBERONC, 37007 Salamanca, Spain; 2 Biochemestry Department, University Hospital of Salamanca, Instituto de Investigación Biomédica de Salamanca (IBSAL), 37007 Salamanca, Spain; 3 Cardiology Department, University Hospital of Salamanca, Instituto de Investigación Biomédica de Salamanca (IBSAL), 37007 Salamanca, Spain; 4 Nephrology Department, University Hospital of Salamanca, 37007 Salamanca, Spain; 5 Pathology Department, University Hospital of Salamanca, 37007 Salamanca, Spain; 6 Hematology Department, University Hospital of Leon, 24008 León, Spain; 7 Internal Medicine Department, University Hospital of Salamanca, 37007 Salamanca, Spain; 8 Pharmacy Department, University Hospital of Salamanca, 37007 Salamanca, Spain

**Keywords:** treatment, daratumumab, AL amyloidosis, response, survival

## Abstract

**Background**: Daratumumab-based regimens represent the gold-standard therapy for newly diagnosed AL amyloidosis patients. However, there are few studies about the efficacy of this treatment in real life. **Methods**: This study included 99 patients: 27 (27.3%) received daratumumab and proteasome inhibitor-based schemes, 46 (46.4%) were treated with proteasome inhibitors and/or immunomodulator-based regimens, and 26 (26.3%) were treated with chemotherapy. **Results**: Patients receiving daratumumab and proteasome inhibitor-based regimens achieved higher rates of partial haematological responses or better (100.0% vs. 78.3% vs. 58.3%; *p* = 0.009 and *p* < 0.001) and complete responses (74.1% vs. 37.0% vs. 12.5%; *p* = 0.003 and *p* < 0.001) than the proteasome inhibitors and/or immunomodulators and chemotherapy groups, respectively. Daratumumab and proteasome inhibitor-based schemes resulted in a shorter time to haematological response (1 month to partial response or better and 4 months to complete response). Moreover, in the group treated with daratumumab and proteasome inhibitor-based regimens, there was a trend of obtaining better and faster organ responses. The benefit of daratumumab and proteasome inhibitor-based regimens was that they resulted in an improvement in progression-free survival (not reached) compared to the proteasome inhibitor and chemotherapy groups (18 months; *p* = 0.022 and 6 months; *p* = 0.002). In addition, the clinical benefit was consistent in patients with Mayo Clinic stages III–IV. **Conclusions**: This study supports the efficacy and superiority of adding daratumumab to the frontline treatment over proteasome inhibitor-based regimens and chemotherapy in AL amyloidosis, including in advanced cardiac disease.

## Simple Summary

The introduction of daratumumab to the initial treatment of patients with AL amyloidosis has been a breakthrough, leading to higher response rates and increased survival. Nevertheless, real-life data on daratumumab in the treatment of newly diagnosed patients are scarce. To date, this is one of the first studies to compare the three treatment milestones in AL amyloidosis, providing real-word evidence of the benefit of adding daratumumab to the frontline therapy in these patients in terms of efficacy and survival.

## 1. Introduction

Immunoglobulin light-chain (AL) amyloidosis is a rare disease caused by the deposition of amyloid fibrils in organ tissues due to misfolded light chains produced by a generally small clone of plasma cells (PCs). It is a hard-to-diagnose disease because of the nonspecificity and heterogeneity of the initial symptoms, resulting in significant diagnosis delays. A high clinical suspicion is crucial for early diagnosis and ameliorates the survival of these patients [1].

AL amyloidosis treatment relies on PC-directed therapies with the aim of achieving rapid and deep haematological responses to slow or intercept amyloid deposition. Historically, AL amyloidosis therapy has followed in the footsteps of multiple myeloma (MM) [2,3].

Oral melphalan in combination with steroids was the backbone treatment for AL amyloidosis for decades [4]. The first main advance started in the mid-1990s with the incorporation of high-dose melphalan and autologous stem cell transplantation (ASCT), which was able to improve overall survival (OS) and became the standard of care for eligible patients [5,6]. Proteasome inhibitors (PIs), particularly bortezomib, were the second milestone, and their combination with cyclophosphamide and dexamethasone (VCD) has been used in first-line treatment for years due to the efficacy and safety [7,8]. The third and probably the most important milestone was the addition of the anti-CD38 monoclonal antibody daratumumab (dara) to VCD, a combination that became the first treatment approved for AL amyloidosis, based on the results of the ANDROMEDA trial [9,10]. In this phase III trial, dara-VCD obtained a significantly greater complete haematological response (hemCR) than the control arm (VCD) (59.5% vs. 19.2%; *p* < 0.001). Also, the addition of dara resulted in significantly greater cardiac (53.0% vs. 24.0%; *p* < 0.001) and renal responses (58.0% vs. 26.0%; *p* < 0.001). These positive outcomes led dara-VCD to become the only approved treatment in 2021 for newly diagnosed AL amyloidosis patients.

Nevertheless, few real-life studies have been conducted to evaluate the use of dara as a frontline therapy in AL amyloidosis [11,12]. Here, to address this gap in the knowledge, we report the results of a real-world study developed in our institution, comparing the efficacies for different treatment milestones of AL amyloidosis in terms of haematological and organ responses, times to responses, and survival.

## 2. Materials and Methods

### 2.1. Patients and Study Design

An observational retrospective study was conducted, and patients with newly diagnosed AL amyloidosis consecutively treated at the University Hospital of Salamanca (Spain) between February 1999 and June 2024 were included. This study was conducted in accordance with the 1964 Declaration of Helsinki and was approved by the ethical committee of the University Hospital of Salamanca.

All patients had biopsy-proven disease by Congo Red staining, with amyloid typing by immunohistochemistry and/or mass spectrometry in doubtful cases. Fluorescence in situ hybridisation (FISH) analysis was performed in 84 patients. FISH was assessed in CD138 positively selected PCs from 2005, and the threshold for positivity was set at 10% for translocations and 20% for chromosomic gains or deletions [13]. High-risk chromosomal abnormalities (HRCAs) were defined as the presence of del(17p) and/or gain(1q). Also, patients were categorised according to the revised Mayo Clinic 2012 prognostic risk model [14].

To evaluate the efficacy of the different treatments used, patients were classified into three groups according to the three treatment milestones of AL amyloidosis: (1) patients receiving dara + PI-based schemes; (2) patients treated with PI and/or immunomodulator (IMiD)-based schemes; and (3) patients receiving chemotherapy (chemo)-based schemes, including in this last group those who directly underwent ASCT. All treatments were administered according to the standard protocols described. This study was designed to assess efficacy, so data regarding safety were not collected.

### 2.2. Response Assessment

Haematological responses were assessed in accordance with the 2012 International Society of Amyloidosis (ISA) criteria [15]. The minimal residual disease (MRD) assessment was performed in patients with suspected hemCR as previously described [16]. Cardiac and renal responses were evaluated according to the graduated response criteria proposed by Muchtar et al. [17,18]. Only patients who were alive (97 patients) were evaluable for the organ response. The time to response and time to hemCR were defined as the time from the start of the treatment until at least a partial response (≥hemPR) or until hemCR, respectively, was achieved.

Progression-free survival (PFS) was defined as the time from diagnosis until clinical progression (haematological and/or organ) or death, whichever occurred first. Overall survival (OS) was defined as the time from diagnosis until the date of death or last follow-up.

### 2.3. Statistical Analysis

The statistical significance of the differences in qualitative and quantitative variables was estimated by the chi-square and the ANOVA tests, respectively. The odds ratio (OR) and 95% confidence interval (CI) were calculated using logistic regression. The Kaplan–Meier method was used to estimate the PFS and OS distributions of the patients. The log-rank test was employed to determine statistically significant differences between the survival of the different subgroups, and the corresponding hazard ratio (HR) and 95% CI were estimated using Cox regression. Values of *p* < 0.05 were considered significant for all statistical tests. Analyses were performed with IBM SPSS Statistics, version 28.

## 3. Results

Ninety-nine patients with newly diagnosed AL amyloidosis were included. Overall, the median age at diagnosis was 64 years (range, 39–90), and 54.5% were men. Amyloid deposits were lambda in most cases (78.8%). The median number of involved organs was 2 (range, 1–6), with the heart (74.7%) and kidney (64.6%) the most frequently affected. Half of the patients were classified as revised Mayo Clinic stages III-IV (54.5%). Only two (2.0%) patients presented concomitant symptomatic MM. We found t(11;14) present in 34.9% of patients, and 8.3% harboured HRCA. The remaining baseline features are shown in Table 1.

We treated 27 patients (27.3%) with dara + PI-based schemes, 46 (46.4%) with PI and/or IMiD-based schemes, and 26 (26.3%) with chemo-based schemes. ASCT was performed as a consolidation treatment in 4 patients (14.8%) of the dara + PI-based scheme group, 18 (39.1%) of the PI and/or IMiD-based scheme group, and 3 (11.5%) of the chemo-based scheme group. Also, in the latter group, nine (34.6%) patients underwent direct ASCT as a frontline therapy. The different treatments used are detailed in Supplementary Figure S1.

Heart involvement was significantly more frequent in the dara + PI-based scheme group (92.6%) compared to the PI and/or IMiD-based (73.9%) or chemo-based scheme groups (57.7%; *p* = 0.014) (Table 1). Notably, more patients treated with dara + PI-based schemes were classified as revised Mayo Clinic stage IV (48.2%) than those treated with PI and/or IMiD-based (31.7%) and chemo-based schemes (10.6%) (*p* = 0.027). Furthermore, patients receiving dara + PI-based schemes were enriched with HRCA (22.2%) compared to the remaining groups (0.0% and 5.5%; *p* = 0.005). No other significant differences were observed among the three groups.

### 3.1. Haematological Response

Haematological responses are summarised in Supplementary Table S1. Seventy-seven (79.4%) patients achieved ≥hemPR and 40 (41.2%) hemCR. The median time to achieve ≥hemPR was 2 months (95% CI, 43.2–78.8) and to hemCR was 18 months (95% CI, not estimable).

**Figure 1:**
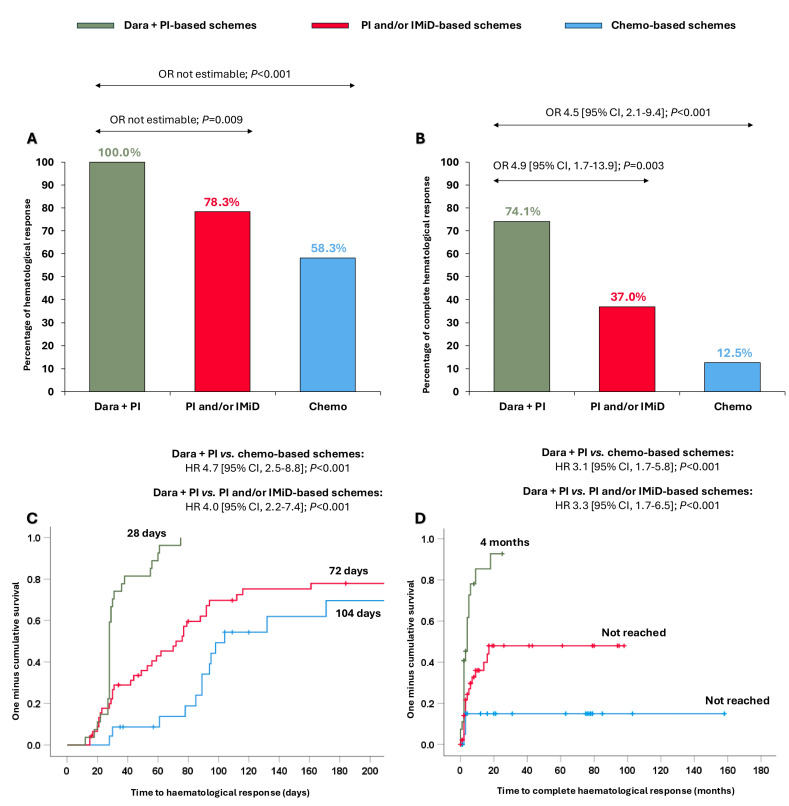
Haematological responses and times to haematological responses in the entire cohort according to the different treatments received. (**A**) Partial haematological response or better; (**B**) complete haematological response; (**C**) time to partial haematological response or better; (**D**) time to complete haematological response. Abbreviations: chemo: chemotherapy; CI: confidence interval; dara: daratumumab; HR: hazard ratio; IMiD: immunomodulators; PI: proteasome inhibitors; OR: odds ratio.

All patients treated with dara + PI-based schemes achieved ≥hemPR (100.0%), which was significantly higher than in those receiving PI and/or IMiD-based schemes (78.3%; OR not estimable, *p* = 0.009) and chemo-based schemes (58.3%; OR not estimable, *p* < 0.001) (Figure 1A). Moreover, 74.1% of patients receiving dara + PI-based-schemes reached hemCR, significantly superior compared to patients treated with PI and/or IMiD-based schemes (37.0%, OR 4.9 [95% CI, 1.7–13.9]; *p* = 0.003) and with chemo-based schemes (12.5%, OR 4.5 [95% CI, 2.1–9.4]; *p* < 0.001) (Figure 1B). Nineteen patients were evaluable for MRD (47.5%), and no differences were observed in MRD negativity among the groups (Supplementary Table S2).

Importantly, these stronger responses were also attained more quickly. The median time to ≥hemPR was significantly shorter with the dara + PI-based schemes (28 days) than with the PI and/or IMiD-based schemes (72 days; HR 4.0 [95% CI, 2.2–7.4]; *p* < 0.001) and with the chemo-based schemes (104 days; HR 4.7 [95% CI, 2.5–8.8]; *p* < 0.001) (Figure 1C). Likewise, the median time to hemCR was significantly shorter in patients receiving dara + PI-based schemes (4 months) than patients receiving PI and/or IMiD-based schemes (not reached; HR 3.3 [95% CI, 1.7–6.5]; *p* < 0.001) and chemo-based schemes (not reached; HR 3.1 [95% CI, 1.7–5.8]; *p* < 0.001) (Figure 1D).

In addition, the same analysis was performed comparing patients treated with dara-VCD (n = 26) and VCD (n = 33). The results, both in terms of haematological response and time to response, were consistent with those described above for dara + PI-based schemes and PI and/or IMiD-based schemes, respectively (Supplementary Figure S2).

### 3.2. Organ Response

Fifty-five (56.7%) patients achieved an organ response, and the median time to a response was 7 months (95% CI, 4.0–10.0). Seventy percent of patients treated with dara + PI-based schemes achieved an organ response, numerically higher than those treated with PI and/or IMiD-based schemes (60.7%; *p* = 0.243) and significantly superior to those treated with chemo-based schemes (41.7%; OR 1.8 [95% CI, 1.1–3.3]; *p* = 0.042) (Supplementary Figure S3A).

Of note, patients who were treated with dara + PI-based schemes obtained an organ response significantly faster (4 months) than patients treated with PI and/or IMiD-based schemes (9 months; HR 2.0 [95% CI, 1.1–3.6]; *p* = 0.029) and chemo-based schemes (19 months; HR 1.6 [95% CI, 1.1–2.4]; *p* = 0.016) (Supplementary Figure S3B).

Cardiac and renal responses according to the criteria proposed by Muchtar et al. are summarised in Supplementary Tables S3 and S4. No differences were observed among the treatment groups in terms of cardiac or renal responses.

As expected, achieving hemCR increased the likelihood of an organ response. More than 80% of patients who were in hemCR achieved an organ response, numerically better in comparison with patients who were in hemVGPR (65.2%, *p* = 0.127), and significantly higher than those who were in hemPR (OR 1.5 [95% CI, 1.5–19.8]; *p* = 0.011) or did not achieve a haematological response (0.0%, OR not estimable; *p* < 0.001). However, no significant differences in the time to organ response were observed among the different categories of haematological response.

### 3.3. Survival Analysis

With a median follow-up of 47 months (range, 2–232), the median PFS and OS of the cohort were 24 months (95% CI, 11.9–36.1) and 95 months (95% CI, 68.7–121.3), respectively. The median follow-ups were 23 months (range, 3–46) in the dara + PI group, 96 months (range, 2–179) in the PI and/or IMiD group, and 105 months (range, 43–232) in the chemo group.

**Figure 2:**
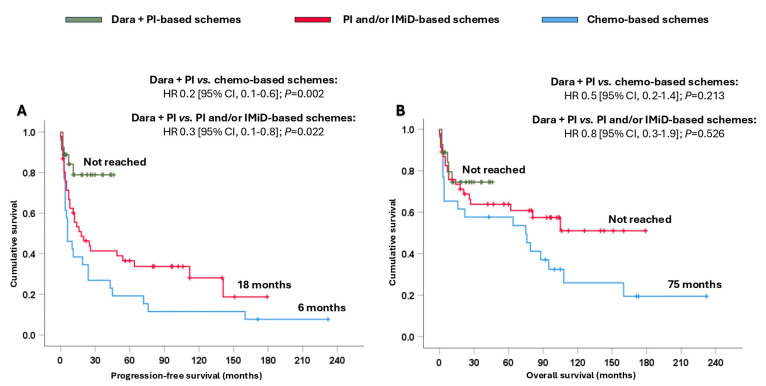
Survival analysis of the entire cohort based on the different treatments received. (**A**) Progression-free survival and (**B**) overall survival. Abbreviations: chemo: chemotherapy; CI: confidence interval; dara: daratumumab; HR: hazard ratio; IMiD: immunomodulators; PI: proteasome inhibitors.

Patients treated with dara + PI-based schemes attained significantly longer PFS (not reached), resulting in a reduction in the likelihood of progression and/or death, ranging from 70% to 80%, compared with patients treated with PI and/or IMiD-based schemes (18 months; HR 0.3 [95% CI, 0.1–0.8]; *p* = 0.022) or chemo-based schemes (6 months; HR 0.2 [95% CI, 0.1–0.6]; *p* = 0.002) (Figure 2A). In terms of OS, remarkable numerical differences were observed among the three groups (Figure 2B).

Moreover, a survival analysis of patients receiving dara-VCD and VCD was performed. The dara-VCD regimen reduced the likelihood of progression and/or death by 60% compared to the VCD regimen (*p* = 0.061). No significant differences in terms of OS between the two arms were observed, probably due to the short follow-up of patients treated with dara-VCD (Supplementary Figure S4).

Rescue treatments in the three arms were also explored: none of the patients treated with dara-VCD had received second-line treatment at the last follow-up. Among the patients treated with PI and/or IMiD-based schemes who progressed, eight (44.4%) received regimens containing dara in the second-line setting. None of the patients receiving chemo in the first line were rescued with dara-based regimens. Since dara was used as a rescue treatment in almost half of the patients of the PI and/or IMiD group, patients were reclassified into dara-exposed and non-exposed to explore the impact of dara on OS. Receiving daratumumab in any line of treatment improved OS compared to those not exposed to dara (105 months vs. 76 months; HR 0.4 [95% CI, 0.2–0.9]; *p* = 0.026) (Supplementary Figure S5).

Fifty-six patients (56.6%) were alive at the last follow-up. No relevant differences were observed in the causes of death among the three groups of treatment, with progression of AL amyloidosis the cause of death in almost half of the cohort (44.2%). The remaining causes of death are shown in Supplementary Table S5.

As expected, achieving hemCR had a positive impact on the survival of AL amyloidosis patients in comparison with the rest of the categories of haematological response (Supplementary Figure S6). Also, an exploratory analysis of survival in patients with MRD assessment is shown in Supplementary Figure S7.

Regarding the effect of an organ response on survival, patients who achieved cardiacCR had a better survival than patients who did not attain cardiacCR, with a median OS not reached (Supplementary Figure S8). In contrast, the degree of renal response had no impact on OS.

### 3.4. Advanced Cardiac Disease (Stages III–IV)

**Figure 3:**
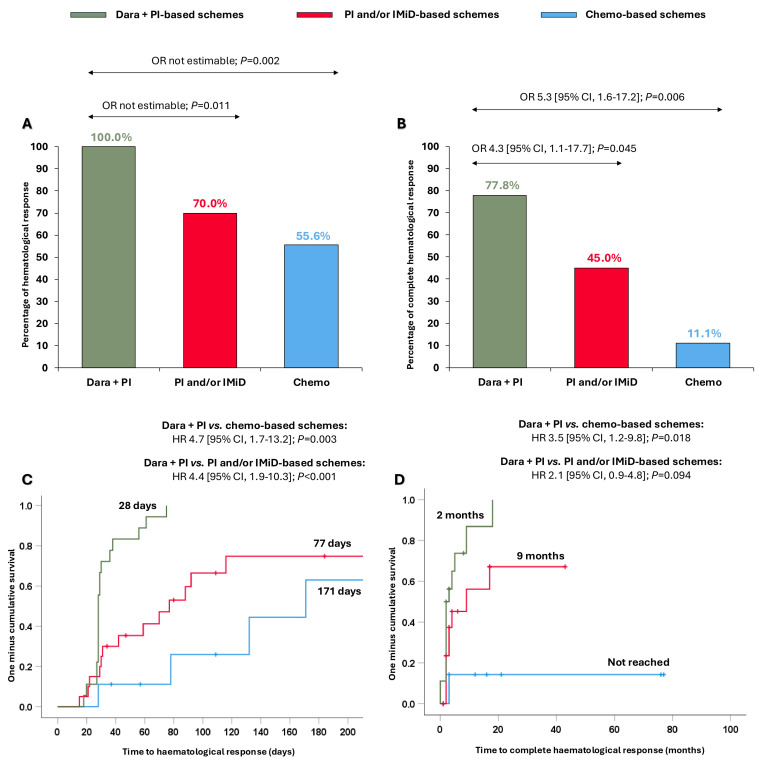
Haematological responses and times to haematological responses in Mayo Clinic stage III-IV patients according to the different treatments received. (**A**) Partial haematological response or better; (**B**) complete haematological response; (**C**) time to partial haematological response or better; and (**D**) time to complete haematological response. Abbreviations: chemo: chemotherapy; CI: confidence interval; dara: daratumumab; HR: hazard ratio; IMiD: immunomodulators; PI: proteasome inhibitors; OR: odds ratio.

We explored whether the clinical benefit observed in the overall cohort with the dara + PI combination was extensible in Mayo stage III-IV patients. Surprisingly, patients treated with dara + PI-based schemes attained ≥hemPR (100.0%), significantly higher than patients treated with PI and/or IMiD-based schemes (70.0%, OR not estimable, *p* = 0.011) and with chemo-based schemes (55.6%, OR not estimable, *p* = 0.002) (Figure 3A). Furthermore, dara + PI-based schemes resulted in significantly superior hemCR (77.8%) compared to the PI and/or IMiD-based schemes (56.5%, OR 4.3 [95% CI, 1.1–17.7]; *p* = 0.045) and the chemo-based schemes (41.7%, OR 5.3 [95% CI, 1.6–17.2]; *p* = 0.006) (Figure 3B). The times to ≥hemPR and hemCR were also quicker with the dara + PI-based schemes in this subset of patients (Figure 3C,D).

Regarding the organ response, 25 patients achieved an organ response (53.2%), with a median time to response of 6 months (95% CI, 2.8–9.2). No significant differences were observed among the groups either in terms of organ response or the time to organ response. However, achieving hemCR also improved the probability of obtaining an organ response. Almost 80% of patients who were in hemCR achieved an organ response, numerically higher than patients who were in hemVGPR (60%, *p* = 0.372) and significantly greater than patients who were in hemPR (37.5%, OR 6.3 [95% CI, 1.1–36.0]; *p* = 0.037) and those who did not achieve haematological response (0.0%, OR not estimable; *p* < 0.001).

**Figure 4:**
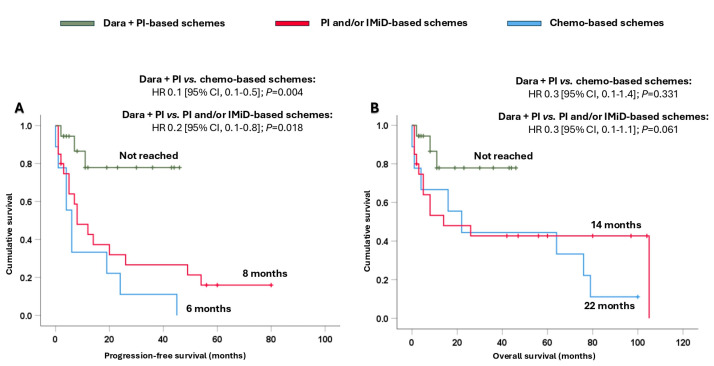
Survival analysis of Mayo Clinic stage III-IV patients based on the different treatments received. (**A**) Progression-free survival and (**B**) overall survival. Abbreviations: chemo: chemotherapy; CI: confidence interval; dara: daratumumab; HR: hazard ratio; IMiD: immunomodulators; PI: proteasome inhibitors.

In addition, it is important to note that the combination of dara + PI significantly prolonged their survival. Thus, the median PFS presented with dara + PI-based schemes was not reached, and it was significantly longer compared to that achieved with PI and/or IMiD-based schemes (8 months, HR 0.2 [95% CI, 0.1–0.8]; *p* = 0.018) and chemo-based schemes (6 months, HR 0.1 [95% CI, 0.1–0.5]; *p* = 0.004) (Figure 4A). Moreover, the combination of dara + PI-based schemes also provided a numerical benefit in terms of OS (Figure 4B).

## 4. Discussion

To our knowledge, this is one of the first studies evaluating and comparing the last three milestones of AL amyloidosis treatment in real-world patients. Our study highlights the effect of the addition of dara to PI in the first line, significantly improving the haematological responses, with 100% ≥hemPR and nearly 75% hemCR. Moreover, this combination resulted in quicker responses (median time to ≥hemPR: 1 month; and median time to hemCR: 4 months) and was key to achieving clinically significant organ responses. In fact, our study confirmed that deeper haematological responses enhanced organ responses. These improved responses led to significantly longer PFS in patients treated with dara + PI-based schemes, with a decrease in the likelihood of progression/death of 70–80% in comparison with PI and/or IMiD-based and chemo-based schemes.

The baseline characteristics of patients in our cohort are consistent with the literature [1,19,20]. Nevertheless, it is important to note that our institution has recently become a reference for cardiac amyloidosis, and that is why the dara + IP-based scheme group was enriched, with more than 90% of patients with cardiac involvement, and more importantly, advanced cardiac disease, with 33% of NYHA III-IV and more than 60% of Mayo Clinic stages III-IV. Despite this, few patients with cardiac stage IIIb were included in our series (11%); therefore, extrapolation of our results to this subset of patients should be performed with caution. Patients with cardiac stage IIIb are unfortunately excluded from most clinical trials, making the management of advanced cardiac AL amyloidosis an unmet medical need. Hopefully, dara as a monotherapy and in combination will be tested in clinical trials, as it could offer a promising option for this difficult-to-treat population [21,22,23].

Patients treated with dara + PI-based schemes achieved outstanding haematological responses: all patients reached ≥hemPR and more than 70% hemCR. Our data are in line with the results reported by the ANDROMEDA trial [9], in which the experimental arm resulted in 90.0% ≥hemPR, 60.0% hemCR, and a median time to ≥hemPR of 2 months. Likewise, superimposable results were reported in the subanalysis of the ANDROMEDA trial in an Asian population [24]. Of note, the control arm (VCD) of both studies mentioned above showed a comparable ≥hemPR (70.0–90.0%) but poorer hemCR (10.0–18.0%) than our PI and/or IMiD-based schemes. One of the potential reasons for this finding is that nearly 40.0% of our patients intensified the response with ASCT. In contrast, our results are slightly better in comparison with the real-world study reported more recently by Bellofiore and colleagues [12]. In that study, 88 newly diagnosed AL amyloidosis patients were treated with dara-based regimens, and 75.0% of patients reached ≥hemPR and 27.0% hemCR at 6 months. These differences could be because, in this series, the patients had a worse performance status (ECOG ≤1: 64.0%), more were at cardiac stage IIIb (26.0%), and patients were more heterogeneously treated. However, both studies agree that dara-based schemes are highly effective in AL amyloidosis, even in advanced-stage disease.

The main goal of the AL amyloidosis treatment, to achieve a rapid and deep haematological response, is crucial to eradicate the amyloidogenic clone and to achieve an organ response. In our study, the addition of dara to PI supported that axiom, improving the organ response and significantly reducing the time to organ response compared to other approaches. Notably, our results are consistent with the cardiac and renal responses shown with dara-VCD in the ANDROMEDA trial (41.5% and 53.0%, respectively) and Asian subanalysis (46.7% and 57.1%, respectively). Similarly, our results were better than those published by Bellofiore and colleagues in terms of cardiac (31.0%) as well as renal responses (26.0%). Furthermore, there was an association between an organ response and hemCR, which was significantly more frequently achieved with dara + PI-based schemes. However, no significant differences were observed between patients who received dara + PI-based schemes and those treated with PI and/or IMiD-based schemes. The factors that hamper organ recovery despite a profound haematological response in AL amyloidosis remain unclear [25,26]. Perhaps, in the future, MRD and mass spectrometry could shed light on why these patients in hemCR do not improve organically [27,28]. In this regard, one of the unmet medical needs in AL amyloidosis is how to improve organ function, especially in those in hemCR. There are clinical trials ongoing to evaluate the efficacy of monoclonal antibodies targeting the amyloid deposits, and if the results are positive, that will become the next milestone in the treatment of this disease [29,30].

As in other haematologic neoplasms, the response is a surrogate marker of survival, and thus, the combination of dara + PI exhibited prolonged median PFS, showing a consistent HR as the latest update of the ANDROMEDA trial [31]. However, only numerical differences were observed in terms of OS, and a longer follow-up is necessary to determine if these differences will be statistically significant. According to other studies, the use of dara, regardless of the line of therapy, results in a significant improvement in OS [23,31,32]. Moreover, as described by other authors, patients who achieved a deep haematological response (CR or ≥VGPR) showed the best outcomes, regardless of the treatment received [15,33]. Despite the clinical relevance of MRD evaluation shown in AL amyloidosis [27], no differences were noted in our series, probably due to the small sample of available assessments. Finally, the role of the organ response in survival is remarkable, especially the cardiac response. In our series, 11.3% patients achieved cardiac CR, all but one were in hemCR, and survival was excellent, as recently described [34]. So, achieving hemCR as well as cardiacCR are a must to overcome a poor prognosis in patients with advanced cardiac disease.

The present study has several limitations. We must highlight its retrospective and observational design, the sample size, and the intrinsically short follow-up of the patients treated with daratumumab. Moreover, since our institution is a referral centre for cardiac amyloidosis, there are inherent biases. In addition, toxicity and patient-reported outcomes were not evaluated. Nevertheless, this study clearly illustrates the history of AL amyloidosis at a centre of excellence in the diagnosis of monoclonal gammopathies, and patients were treated in accordance with the current standard of care at the time.

## 5. Conclusions

In conclusion, this study provides real-world evidence of the benefit of the incorporation of dara into the frontline setting for treatment of AL amyloidosis. The addition of dara to PI allows for rapid and deep haematological and organ responses, and subsequently results in prolonged survival, even in those with advance cardiac disease. The incorporation of dara into PI in the frontline treatment represents the new paradigm shift that is changing the natural course of AL amyloidosis.

## Figures and Tables

**Table 1:** Baseline characteristics of the entire cohort.

	Entire Cohort (N = 99)	Dara + PI-Based Schemes (n = 27)	PI and/or IMiD-Based Schemes (n = 46)	Chemo-Based Schemes (n = 26)	*p* Value
Age of diagnosis, median (range)	64 (39–90)	56 (44–82)	63 (40–89)	66 (39–90)	0.732
≥60 years, n (%)	55 (55.6)	13 (48.1)	26 (56.5)	16 (61.5)	0.608
Gender male, n (%)	54 (54.5)	15 (55.6)	22 (47.8)	8 (30.8)	0.176
ECOG 0–1, n (%)	70 (70.7)	21 (77.8)	34 (73.9)	15 (57.7)	0.222
Time between symptomatology onset and diagnosis, median (range)	5 (1–48)	4.5 (2–13)	6 (1–48)	3 (1–36)	0.315
Organ involvement, median (range)	2 (1–6)	2 (1–3)	2 (1–6)	2 (1–4)	0.996
Heart, n (%)	74 (74.7)	25 (92.6)	34 (73.9)	15 (57.7)	0.014
Renal, n (%)	64 (64.6)	17 (63.0)	28 (60.9)	19 (73.1)	0.569
Peripheral neuropathy, n (%)	23 (23.2)	5 (18.5)	11 (23.9)	7 (26.9)	0.761
Gastrointestinal tract, n (%)	17 (17.2)	4 (14.8)	7 (15.2)	6 (23.1)	0.648
Liver, n (%)	11 (11.2)	3 (11.1)	4 (8.7)	4 (15.4)	0.686
Autonomic nervous s., n (%)	8 (8.1)	2 (7.4)	4 (8.7)	2 (7.7)	0.978
Periorbital purpura, n (%)	8 (8.1)	3 (11.1)	3 (6.5)	2 (7.7)	0.783
Macroglossia, n (%)	4 (4.1)	0 (0.0)	4 (0.7)	0 (0.0)	0.091
Lambda free light chain, n (%)	78 (78.8)	22 (81.4)	37 (80.4)	19 (73.1)	0.705
dFLC (mean ± SD)	848.0 ± 3731.1	1711.1 ± 6808.0	538.9 ± 1100.8	415.6 ± 576.1	0.346
Immunoparesis, n (%)	68 (68.7)	21 (77.8)	30 (65.2)	17 (65.3)	0.524
Revised Mayo Clinic, n (%)					
Stage I	14/87 (16.1)	2/27 (7.4)	7/41 (17.1)	5/19 (26.3)	0.222
Stage II	26/87 (29.9)	7/27 (25.9)	14/41 (34.1)	5/19 (26.3)	0.714
Stage III	19/87 (21.8)	5/27 (18.5)	7/41 (17.1)	7/19 (36.8)	0.199
Stage IV	28/87 (32.2)	13/27 (48.2)	13/41 (31.7)	2/19 (10.6)	0.027
NT-proBNP, pg/mL (mean ± SD)	3770.6 ± 4175.1	3604.2 ± 3232.6	4149.9 ± 4162.1	3140.0 ± 3961.1	0.622
≥NT-proBNP 8500 pg/mL, n (%)	12/95 (12.6)	3 (11.1)	7 (15.2)	2/22 (9.1)	0.746
NYHA III-IV, n (%)	25 (25.3)	9 (33.3)	9 (19.6)	5 (19.2)	0.524
Cardiac stage IIIb, n (%)	11/87 (12.6)	3 (11.1)	6/41 (14.6)	2/19 (10.5)	0.673
Proteinuria > 5 g/day, n (%)	35 (35.4)	12 (44.4)	13 (28.3)	10 (38.5)	0.369
Albumin, g/dL (mean ± SD)	3.1 ± 0.9	3.1 ± 0.9	3.2 ± 1.0	2.9 ± 0.7	0.493
Alkaline phosphatase, U/L (mean ± SD)	146.2 ± 281.9	103.5 ± 67.2	127.4 ± 153.5	228.3 ± 108.7	0.244
≥10% PC in BM, n (%)	34 (34.3)	8 (29.6)	18 (39.1)	8 (30.7)	0.594
MM associated, n (%)	2 (2.0)	1 (3.7)	0 (0.0)	1 (3.8)	0.412
Cytogenetic abnormalities, n (%)					
t(11;14)	29/83 (34.9)	9/27 (33.3)	15/37 (40.5)	5/19 (26.3)	0.559
High risk *	7/84 (8.3)	6/27 (22.2)	0/38 (0.0)	1/19 (5.3)	0.005

Abbreviations: BM: bone marrow; chemo: chemotherapy; dara: daratumumab; dFLC: difference between involved and uninvolved serum-free light chains; ECOG: European Cooperative Oncology Group; IMiD: immunomodulators; MM: multiple myeloma; NYHA: New York Heart Association; PC: plasma cell; PI: proteasome inhibitors; SD: standard deviation. * High-risk cytogene were defined as del17p and/or 1q.

## Data Availability

Due to the sensitive nature of the data, information created during and/or analysed during the current study is available from the first and corresponding authors.

## Supplementary Materials

The following supporting information can be downloaded at https://www.mdpi.com/article/10.3390/cancers17091440/s1, Figure S1: Regimens used as first-line treatments in the entire cohort. Table S1: Haematological responses in the entire cohort and according to the different treatments received. Table S2: Minimal residual disease assessment in patients who achieved a complete haematological response according to the different treatments received. Figure S2: Haematological responses and times to haematological responses in patients receiving Dara-VCD and VCD. Figure S3: Organ responses in the entire cohort according to the different treatments received. Table S3: Cardiac responses in the entire cohort and according to the different treatments received. Table S4: Renal responses in the entire cohort and according to the different treatments received. Table S5: Causes of death in the entire cohort and by the different treatments received. Figure S4: Survival analysis in patients receiving Dara-VCD and VCD. Figure S5: Analysis of overall survival across the entire cohort based on daratumumab treatment in any line of therapy. Figure S6: Survival analysis of the entire cohort based on the different haematological responses achieved. Figure S7: Impact of minimal residual disease on survival in patients who achieved a complete haematological response. Figure S8: Survival analysis in the entire cohort according to the different graded cardiac responses.
