# Supplementary material for: Impact of the Addition of Daratumumab to the Frontline Treatment of Patients with Immunoglobulin Light-Chain Amyloidosis: A Single-Centre Experience
Source: Cancers (Basel). 2025 Apr 25;17(9):1440. doi: 10.3390/cancers17091440 (PMC12071125; doi:10.3390/cancers17091440)
Supplement: Supplementary file 1 [file cancers-17-01440-s001.zip › Supplementary material.pdf]

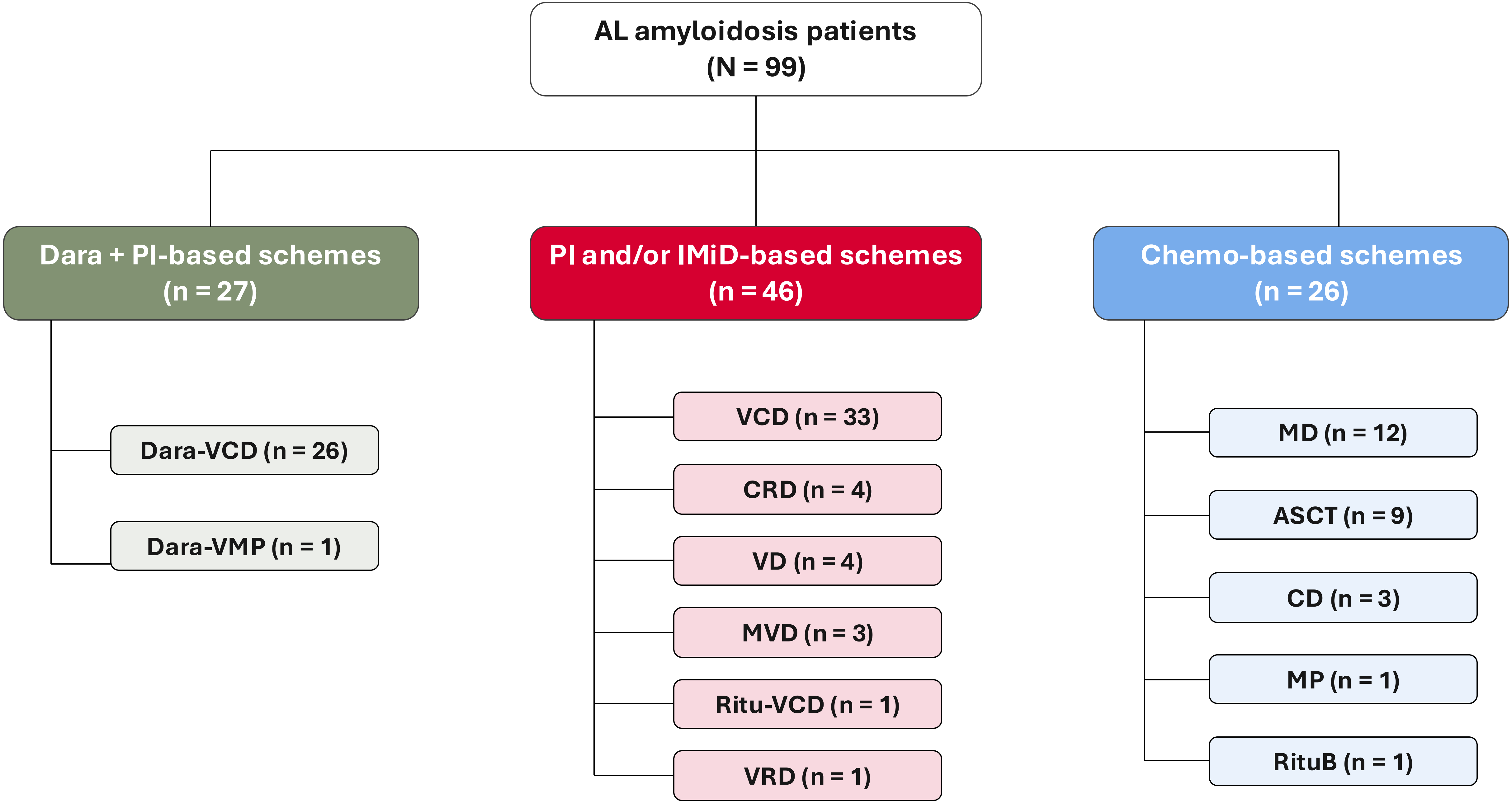

Supplementary Figure S1. Regimens used as first-line treatments in the global cohort. Abbreviations: ASCT: autologous stem cell transplantation; B: bendamustine; C: cyclophosphamide; chemo: chemotherapy; D: dexametasone; dara: daratumumab; IMiD: immunomodulators; M: melphalan; P: prednisone; PI: proteasome inhibitors; R: lenalidomide; Ritu: Rituximab; V: bortezomib.

Supplementary Table S1. Haematological responses in the entire cohort and by the different treatments received.

	**Entire cohort (N=97)**	**Dara + PI-based schemes (n=27)**	**PI and/or IMiD-based schemes (n=46)**	**Chemo-based schemes (n=24)**	***P* value**
CR, n (%)	40 (41.2)	20 (74.1)	17 (37.0)	3 (12.5)	**< 0.001**
VGPR, n (%)	23 (23.7)	3 (11.1)	11 (23.9)	9 (37.5)	0.087
PR, n (%)	14 (14.4)	4 (14.8)	8 (17.4)	2 (8.3)	0.591
SD, n (%)	4 (4.1)	0	1 (2.2)	3 (12.5)	0.053
PD, n (%)	16 (16.5)	0	9 (19.5)	7 (29.2)	**0.015**

Abbreviations: chemo: chemotherapy; CR: complete response; dara: daratumumab; IMiD: immunomodulators; PD: progression disease; PI: proteasome inhibitors; PR: partial response; SD: stable disease; VGPR: very good partial response.

Supplementary Table S2. Minimal residual disease assessment in patients who achieved haematological complete response by the different treatments received.

	**Entire cohort (N=19)**	**Dara + PI-based schemes (n=9)**	**PI and/or IMiD-based schemes (n=9)**	**Chemo-based schemes (n=1)**	***P* value**
MRD negative, n (%)	12 (63.2)	6 (66.7)	5 (55.6)	1 (100.0)	0.383
MRD positive, n (%)	7 (36.8)	3 (33.3)	4 (44.4)	0 (0)	

Abbreviations: chemo: chemotherapy; dara: daratumumab; IMiD: immunomodulators; MRD: minimal residual disease; PI: proteasome inhibitors.

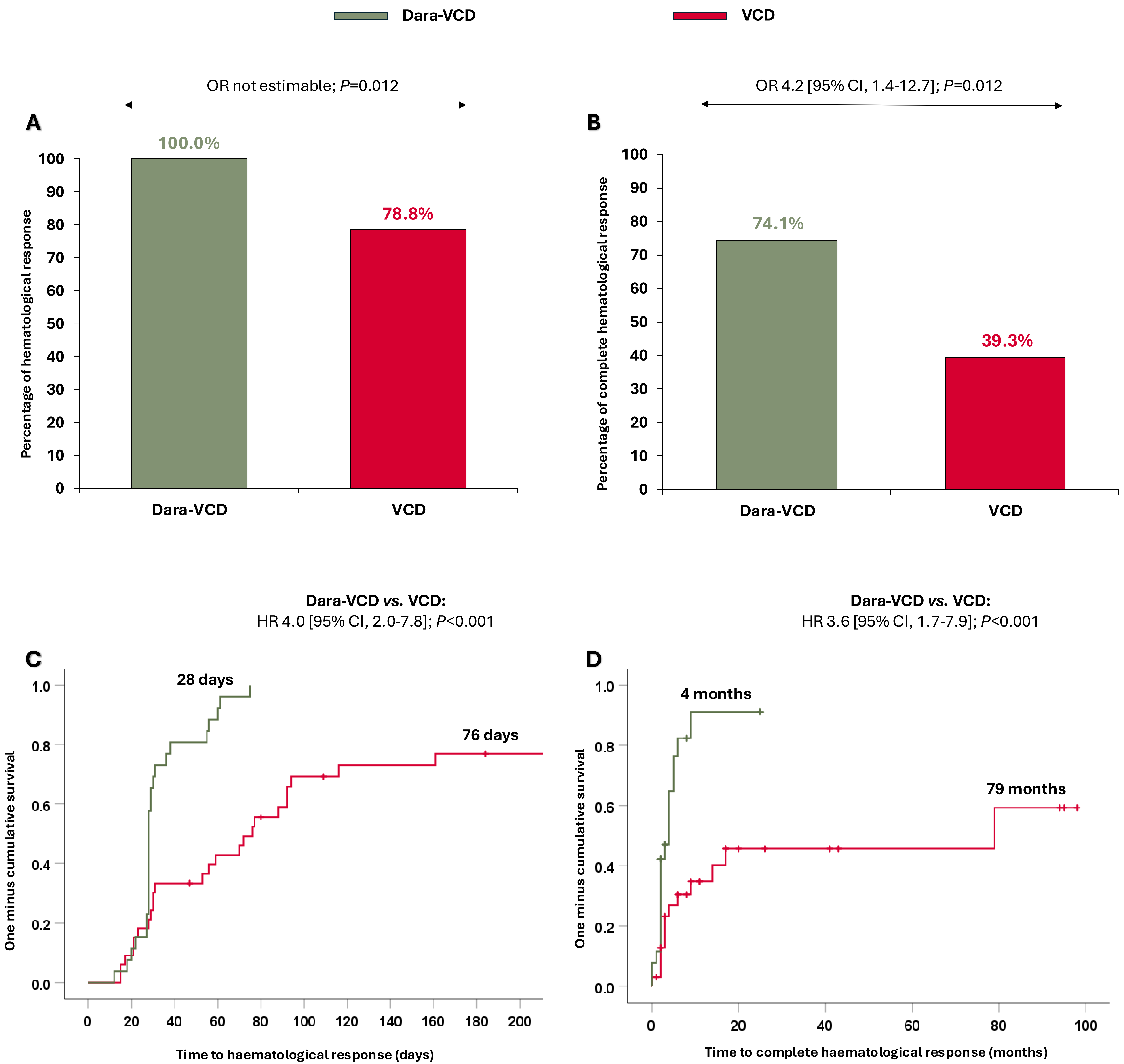

Supplementary Figure S2. Haematological responses and time to haematological response in patients receiving Dara-VCD and VCD. **A)** Partial haematological response or better; **B)** complete haematological response; **C)** time to partial haematological response or better; **D)** time to complete haematological response. Abbreviations: CI: confidence interval; dara: daratumumab; HR: hazard ratio; OR: odds ratio; VCD: bortezomib, cyclophosphamide, dexamethasone.

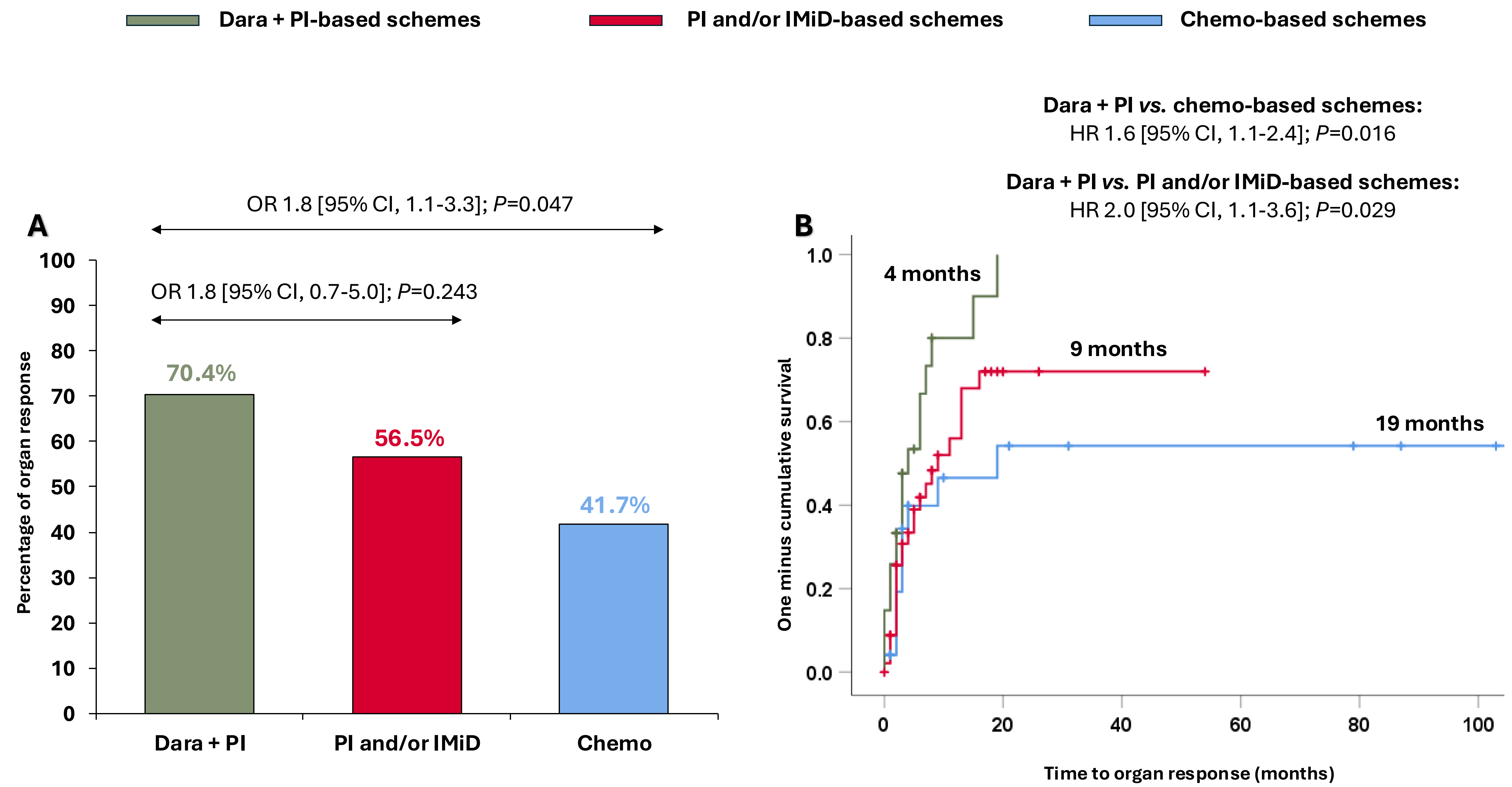

Supplementary Figure S3. Organ responses in the entire cohort by the different treatments received. **A)** Partial organ response or better and **B)** time to organ response. Abbreviations: chemo: chemotherapy; CI: confidence interval; dara: daratumumab; HR: hazard ratio; IMiD: immunomodulators; PI: proteasome inhibitors; OR: odds ratio.

Supplementary Table S3. Cardiac responses in the entire cohort and by the different treatments received.

	**Entire cohort (N=71)**	**Dara + PI-based schemes (n=25)**	**PI and/or IMiD-based schemes (n=34)**	**Chemo-based schemes (n=12)**	***P* value**
CR, n (%)	8 (11.3)	4 (16.0)	3 (8.8)	1 (8.3)	0.648
VGPR, n (%)	10 (14.1)	4 (16.0)	4 (11.8)	2 (16.7)	0.864
PR, n (%)	16 (22.5)	6 (24.0)	10 (29.4)	0 (0.0)	0.108
NR, n (%)	37 (52.1)	11 (44.0)	17 (50.0)	9 (75.0)	0.198

Abbreviations: chemo: chemotherapy; CR: complete response; dara: daratumumab; IMiD: immunomodulators; NR: no response; PI: proteasome inhibitors; PR: partial response; VGPR: very good partial response.

Supplementary Table S4. Renal responses in the entire cohort and by the different treatments received.

	**Entire cohort (N=61)**	**Dara + PI-based schemes (n=17)**	**PI and/or IMiD-based schemes (n=28)**	**Chemo-based schemes (n=16)**	***P* value**
CR, n (%)	7 (11.5)	3 (17.6)	2 (7.1)	2 (12.5)	0.557
VGPR, n (%)	15 (24.6)	7 (41.2)	6 (21.4)	2 (12.5)	0.140
PR, n (%)	15 (24.6)	3 (17.6)	9 (32.1)	3 (18.7)	0.450
NR, n (%)	24 (39.3)	4 (23.6)	11 (29.3)	9 (56.3)	0.157

Abbreviations: chemo: chemotherapy; CR: complete response; dara: daratumumab; IMiD: immunomodulators; NR: no response; PI: proteasome inhibitors; PR: partial response; VGPR: very good partial response.

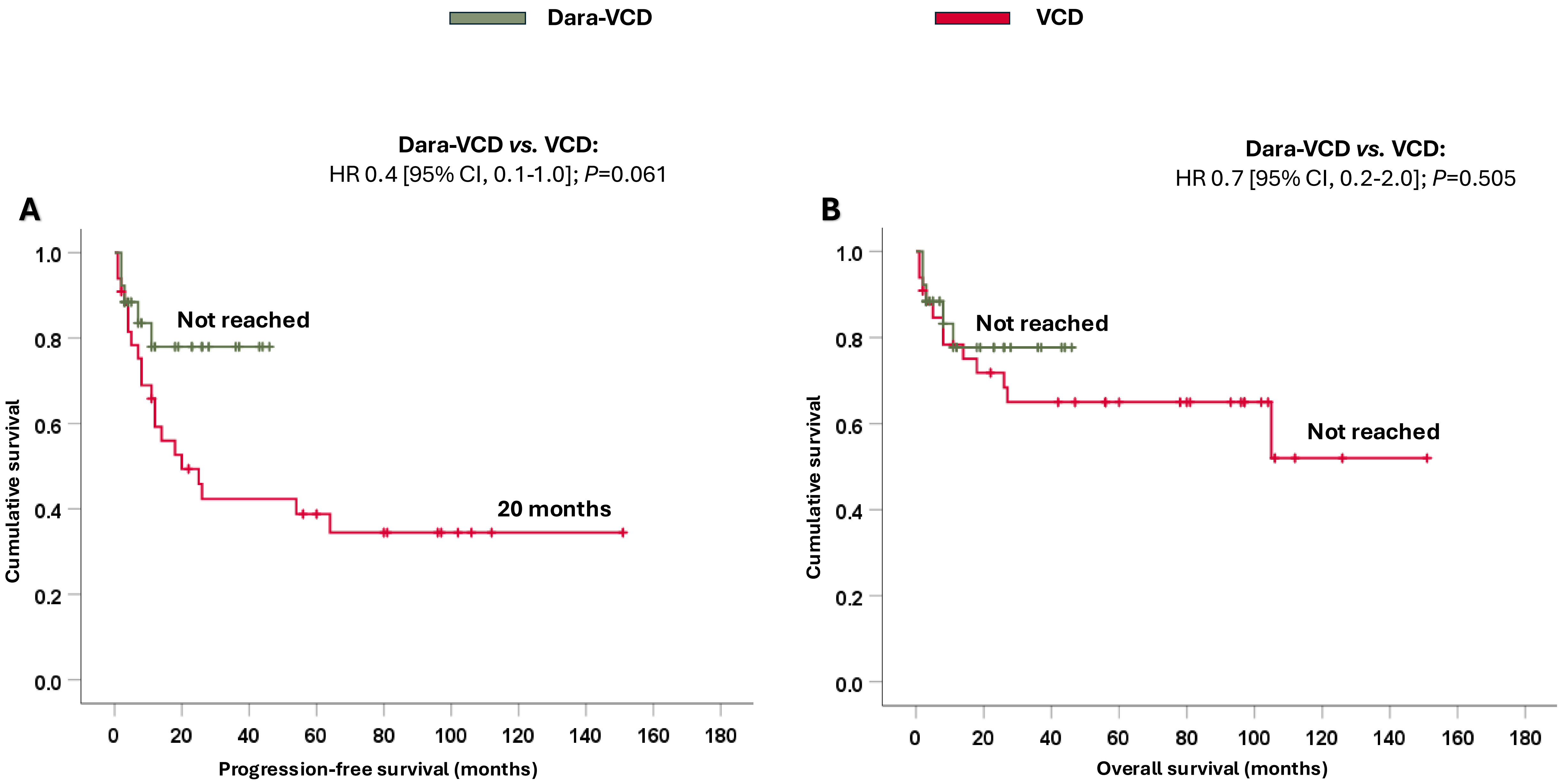

Supplementary Figure S4. Survival analysis in patients receiving Dara-VCD and VCD. **A)** Progression-free survival and **B)** overall survival. Abbreviations: CI: confidence interval; dara: daratumumab; HR: hazard ratio; VCD: bortezomib, cyclophosphamide, dexamethasone.

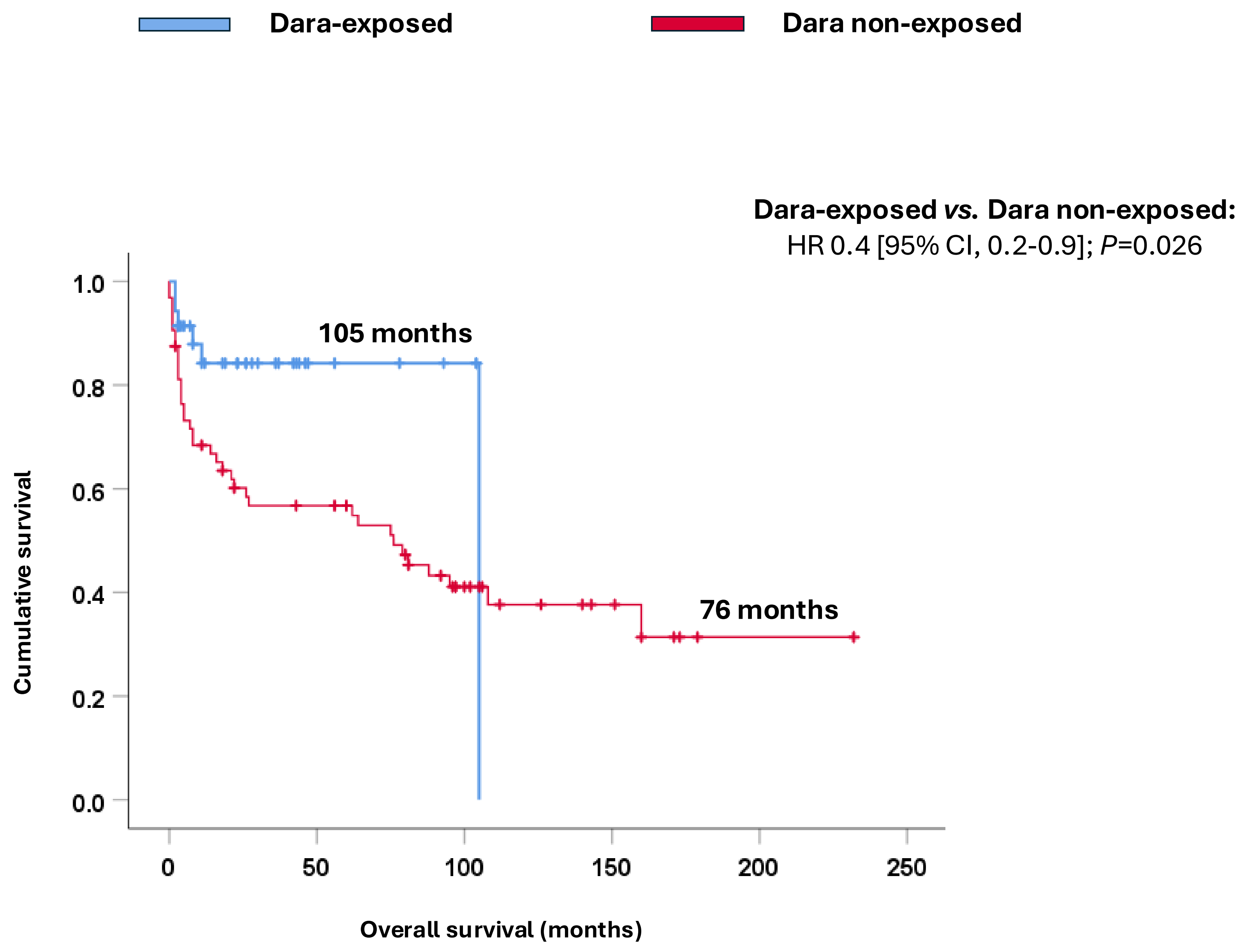

Supplementary Figure S5. Analysis of overall survival across the entire cohort based on daratumumab treatment in any line of therapy. Abbreviations: CI: confidence interval; Dara: daratumumab; HR: hazard ratio.

Supplementary Table S5. Causes of death in the entire cohort and by the different treatments received.

	**Entire cohort (N=43)**	**Dara + PI-based schemes (n=5)**	**PI and/or IMiD-based schemes (n=19)**	**Chemo-based schemes (n=19)**
Related with AL amyloidosis, n (%)				
Progression	19 (44.2)	2 (40.0)	7 (36.8)	10 (52.6)
Related with treatment, n (%)				
Infections	14 (32.6)	1 (20.0)	7 (36.8)	6 (31.6)
Cardiac transplant failure	1 (2.3)	0 (0.0)	1 (5.3)	0 (0.0)
Progression to other hematological malignancies	1 (2.3)	0 (0.0)	1 (5.3)	0 (0.0)
No related, n (%)				
Other malignancies	4 (9.3)	1 (20.0)	1 (5.3)	2 (10.5)
Stroke	1 (2.3)	1 (20.0)	0 (0.0)	0 (0.0)
Unknown, n (%)	3 (7.0)	0 (0.0)	2 (10.5)	1 (5.3)

Abbreviations: chemo: chemotherapy; dara: daratumumab; IMiD: immunomodulators; PI: proteasome inhibitors.

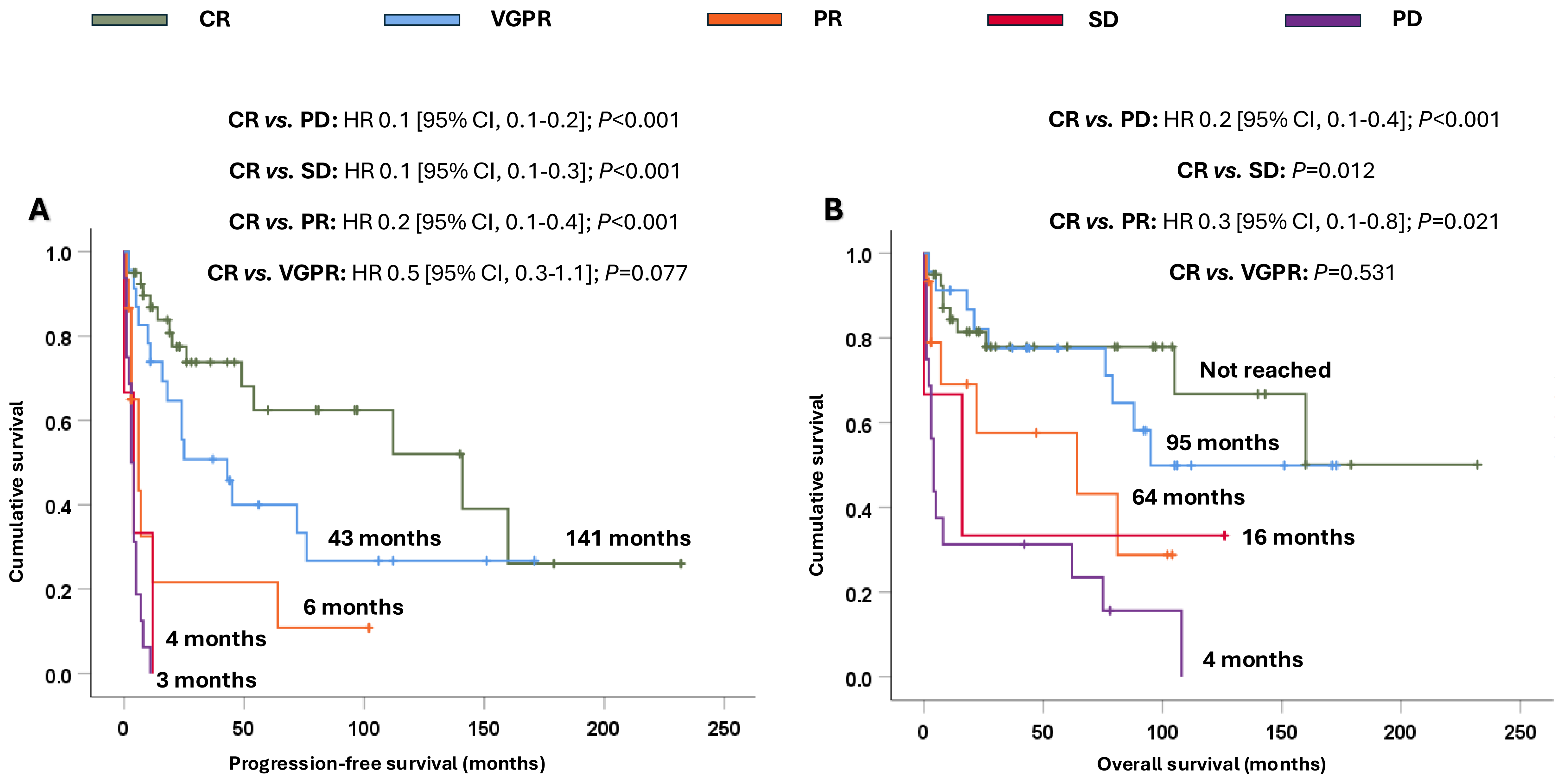

Supplementary Figure S6. Survival analysis of the entire cohort based on the different hematological responses achieved. **A)** Progression-free survival and **B)** overall survival. Abbreviations: CI: confidence interval; CR: complete response; HR: hazard ratio; PD: progression disease; PR: partial response; SD: stable disease; VGPR: very good partial response.

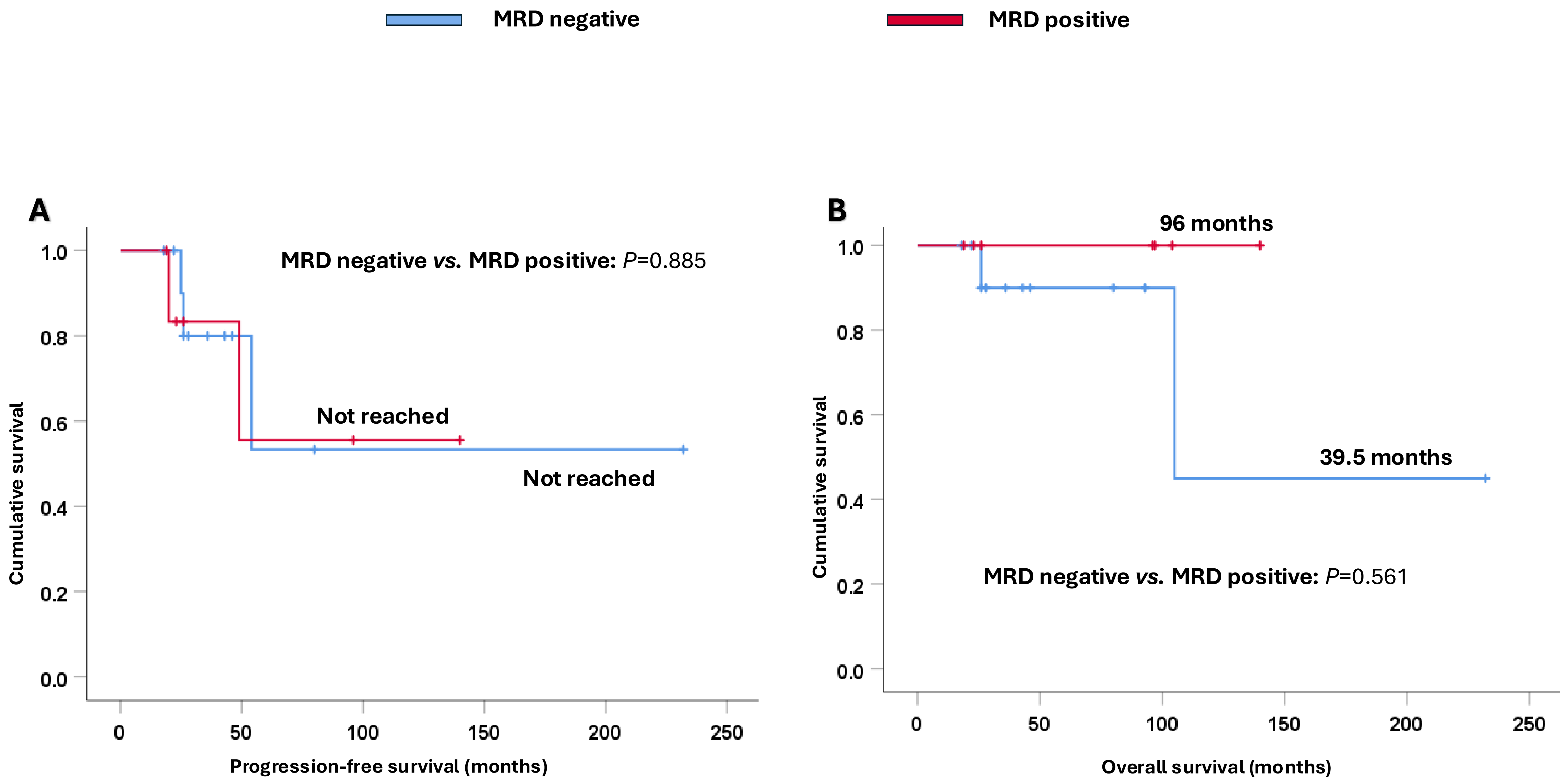

Supplementary Figure S7. Impact of minimal residual disease on survival in patients who achieved hematological complete response. **A)** Progression-free survival and **B)** overall survival. Abbreviations: MRD: minimal residual disease.

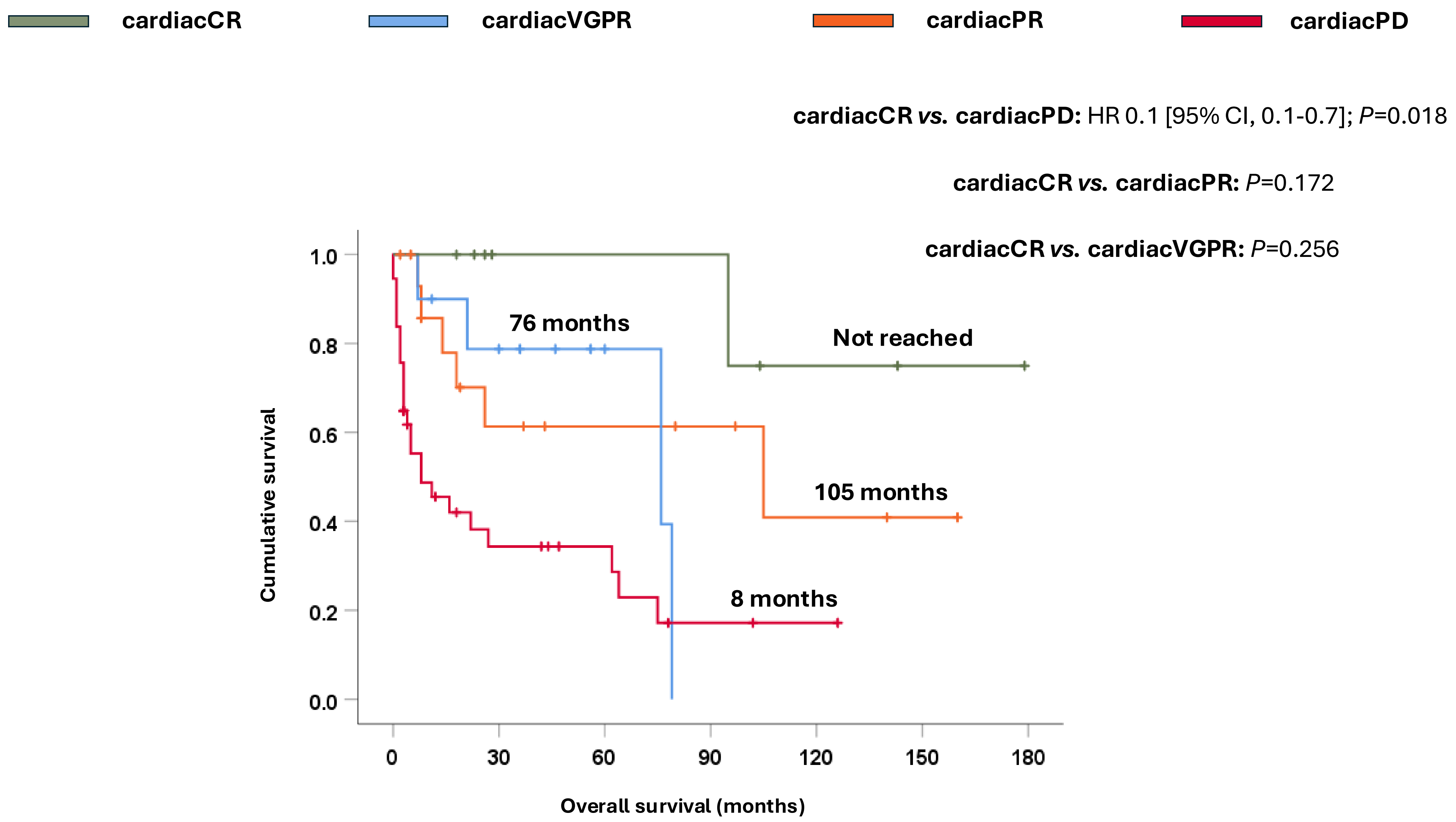

Supplementary Figure S8. Overall survival analysis in the entire cohort by the different graded cardiac responses. Abbreviations: CI: confidence interval; CR: complete response; HR: hazard ratio; PD: progression disease; PR: partial response; VGPR: very good partial response.
